# Comparative analysis of sucrose phosphate synthase (SPS) gene family between *Saccharum officinarum* and *Saccharum spontaneum*

**DOI:** 10.1186/s12870-020-02599-7

**Published:** 2020-09-14

**Authors:** Panpan Ma, Xingtan Zhang, Lanping Chen, Qian Zhao, Qing Zhang, Xiuting Hua, Zhengchao Wang, Haibao Tang, Qingyi Yu, Muqing Zhang, Ray Ming, Jisen Zhang

**Affiliations:** 1 Center for Genomics and Biotechnology, Haixia Institute of Science and Technology, Fujian Provincial Key Laboratory of Haixia Applied Plant Systems Biology, College of Crop Science, Fujian Agriculture and Forestry University, Fuzhou, 350002 China; 2 Institute of Applied Ecology, Fujian Agriculture and Forestry University, Fuzhou, 350002 China; 3 College of Life Sciences, Fujian Normal University, Fuzhou, 350007 China; 4 Texas A&M AgriLife Research, Department of Plant Pathology and Microbiology, Texas A&M University System, Dallas, TX 75252 USA; 5 Guangxi Key Lab of Sugarcane Biology, Guangxi University, Nanning, Guangxi China; 6 Department of Plant Biology, University of Illinois at Urbana-Champaign, Urbana, IL 61801 USA

**Keywords:** Sugarcane, *S. officinarum*, *S. spontaneum*, Sucrose phosphate synthase (SPS), Polyploidy, BAC libraries, Transcriptome, Metabolites

## Abstract

**Background:**

Sucrose phosphate synthase (SPS) genes play vital roles in sucrose production across various plant species. Modern sugarcane cultivar is derived from the hybridization between the high sugar content species *Saccharum officinarum* and the high stress tolerance species *Saccharum spontaneum*, generating one of the most complex genomes among all crops. The genomics of sugarcane SPS remains under-studied despite its profound impact on sugar yield.

**Results:**

In the present study, 8 and 6 gene sequences for SPS were identified from the BAC libraries of *S. officinarum* and *S. spontaneum*, respectively. Phylogenetic analysis showed that *SPSD* was newly evolved in the lineage of Poaceae species with recently duplicated genes emerging from the *SPSA* clade. Molecular evolution analysis based on Ka/Ks ratios suggested that polyploidy reduced the selection pressure of *SPS* genes in *Saccharum* species. To explore the potential gene functions, the *SPS* expression patterns were analyzed based on RNA-seq and proteome dataset, and the sugar content was detected using metabolomics analysis. All the *SPS* members presented the trend of increasing expression in the sink-source transition along the developmental gradient of leaves, suggesting that the *SPSs* are involved in the photosynthesis in both *Saccharum s*pecies as their function in dicots. Moreover, *SPSs* showed the higher expression in *S. spontaneum* and presented expressional preference between stem (*SPSA*) and leaf (*SPSB*) tissue, speculating they might be involved in the differentia of carbohydrate metabolism in these two *Saccharum* species, which required further verification from experiments.

**Conclusions:**

*SPSA* and *SPSB* genes presented relatively high expression and differential expression patterns between the two *Saccharum* species, indicating these two *SPSs* are important in the formation of regulatory networks and sucrose traits in the two *Saccharum* species. *SPSB* was suggested to be a major contributor to the sugar accumulation because it presented the highest expressional level and its expression positively correlated with sugar content. The recently duplicated *SPSD2* presented divergent expression levels between the two *Saccharum* species and the relative protein content levels were highest in stem, supporting the neofunctionalization of the *SPSD* subfamily in *Saccharum*.

## Background

Sucrose is produced in plant leaves following photosynthesis along with other carbohydrates. The key organic compound constitutes the most abundant form of soluble storage carbohydrate, which can be utilized directly by glycolysis or transported from photosynthetic tissues to non-photosynthetic tissues via the phloem [1]. Sucrose therefore serves as a source of fixed carbon that can be distributed systemically throughout the plant, providing fundamental resources for direct energy production or biosynthesis of long chains of biopolymers such as starch [2] and cellulose [3].

Sucrose is synthesized in the cytosol, starting with the export of dihydroxyacetone phosphate and glyceraldehyde phosphate from the chloroplast. The following processes are catalyzed by a series of enzymes [4], in which sucrose phosphate synthase (SPS) is one of the most important ones. SPS catalyzes the conversion of Fructose-6-Phosphate (F-6-P) and UDP-Glucose (UDP-G) to Sucrose-6-Phosphate (S-6-P), providing the substrates for sucrose phosphate phosphatase (SPP). In the final step, sucrose is generated through the removal of the phosphate group. In addition to the well-recognized role of SPS in sucrose biosynthesis in source leaves, it is becoming clear that SPS also plays an important and key role in heterotrophic cells engaging in the net sucrose degradation [5]. For example, significant turnover of the endogenous sucrose pool was observed in germinating *Ricinus* cotyledons [6]. This turnover of sucrose is thought to be involved in a futile cycle of simultaneous synthesis and cleavage, resulting from changes in the activation rate of SPS phosphorylation [7]. Therefore, SPS plays a crucial role in carbohydrate metabolism by regulating the partitioning of carbon between starch production and carbohydrate (sucrose) accumulation in many physiological and developmental processes.

The role of SPS was first demonstrated in wheat germ extracted by Leloir and Cardini [8] and some plants had multiple *SPS* genes and expression of these copies varies with developmental stages, tissue types and environmental signals [9–12], suggesting that *SPS* genes played divergent roles under different conditions. Recent studies showed that most *SPS* genes were clustered into three distinct families (A, B and C) and these genes appear to have different evolutionary histories in dicots (A family) and monocots (B family) [4]. Even though one of the *SPS* isoforms from sugarcane and its closely related partial sequence from barley were grouped in a family, they were somewhat more divergent than the remaining dicot *SPSs* that have been characterized [4]. SPS transformation experiments indicated that SPS was a major determinant inpartitioning fixed carbon from photosynthesis in the leaf and in the whole plant [13, 14]. Recently, Mark simultaneously increased SPS and glutamine synthetase (GS) activities in transgenic tobacco and found that sucrose was the major determinant of growth and development [15].

Sugarcane is the most important sugar crop in the world since it accounts for 80% worldwide sugar yield [16]. Previous SPS studies in sugarcane showed that the SPS activity and transcript expression showed higher in mature internodes than in immature internodes for all studied cultivars [17]. Meanwhile, compared to the low sugar species of sugarcane, the high sugar species showed increased transcript expressions and enzyme activities of SPS at all developmental stages [17]. In addition, expression of SPS decreased significantly in the late maturing sugarcane variety BO91, compared to the early maturing sugarcane variety CoJ64 [18]. The SPS members were predicted based on public EST databases [19, 20], and DNA fragments of amplified products from Q165 and IJ76–514 cultivars have been characterized to allow the identification all possible alleles [19, 20]. Recent research showed that the N-terminal region of sugarcane SPS played an important role in allosteric regulation [21]. Despite the profound and well-documented role of SPS in sugarcane, the genomic sequences and biological functions for SPS members have not been identified in sugarcane due to their complicated genomes. *S. officinarum* and *S. spontaneum* are two of the most important *Saccharum* species not only because they are the major contributors to the genomes of modern sugarcane varieties, but they are also quite divergent with respect to sugar production [22]. In this study, to comprehensively characterize the *SPS* family at the molecular and evolutionary level as well as the possible functions of the *SPS* family in the two main *Saccharum* species, we analyzed the *SPS* gene family in *S. officinarum* and *S. spontaneum* and illustrated their evolutionary history, the structural and expressional differences as well possible regulatory factors through the utilization of the combinatorial analysis of transcriptome, metabolome and proteome data.

## Results

### Identification of *SPS* gene family in *S. officinarum* and *S. spontaneum*

LA Purple (*S. officinarum*, 2n = 80) and AP85–441 (the haploid clone of SES208, 2n = 4x = 32) derived from the anther culture of SES208 (*S. spontaneum*, 2n = 64) [23] representing two major *Saccharum* species were used for the construction of the BAC library. Eight and six *SPS*-containing BACs were isolated from *S. officinarum* and *S. spontaneum*, respectively (Table 1), with an average length of 68.6 kb, and maximum length of 127.2 kb (BAC id: SES23E05). TE annotation suggested that Long Terminal Repeats (LTRs) were the major repetitive sequences in most of isolated BACs (Table 1). The putative genes including 14 sugarcane *SPS* sequences were annotated from the selected BACs (Additional file 1). Among the 14 *SPS* genes, 11 contained complete ORFs (open reading frames), with the length of coding sequences ranging from 1404 bp to 3321 bp. For further validation, these putative *SPS* genes were blasted against *Sorghum SPS* genes and they showed high similarity, with identities ranging from 91 to 100% at the amino acid level (Table 2). In this study, we refer to the sugarcane *SPS* genes using *SPSA* to *SPSD* according to the sequence similarity with *Sorghum SbSPSs* with a prefix ‘So’ for *S. officinarum* and ‘Ss’ for *S. spontaneum*. We also identified *SPS* genes from the recently published *Saccharum spontanenum* genome [24]. Five genes without alleles were found with a similarity above 91% compared to 6 BAC sequences (Additional file 2). There were three genes of *SPSD2* (*Sspon.004A0021251, Sspon.004A0021261* and *Sspon.004A0021270*) in the *S. spontaneum* genome. To identify the duplication of three *SPSD2* genes, a MCScanX program was used to the analysis referring to the research of Wang et al. [25]. The results indicated that *Sspon.004A0021251* may be the primary gene produced from the whole genome duplications (WGDs), and *Sspon.004A0021261* and *Sspon.004A0021270* were two genes from the tandem duplication. Furthermore, to strengthen the reliability of the sequences, *Sspon.007C0001731, Sspon.004A0021270* and *Sspon.004A0021251* were re-annotated (Additional file 3).

**Table 1:** The results of the repeat sequence annotation for BACs containing *SPS*

Specie	BAC ID	Probe	Transposable elements (%)			Tandem repeat sequence (%)		
			LTR	Non-LTR	Transposons	SSR	Satellite	Low complexity
*S. officinarum* (LA Purple)	84F06	*SPSA*	30.64	2.62	0.45	1.41	0.00	0.00
	34B02	*SPSB*	15.55	1.74	3.71	1.24	0.00	0.17
	154P24	*SPSB*	19.59	2.13	1.61	1.25	0.00	0.13
	33C13	*SPSC*	15.83	0.10	0.94	1.62	0.00	0.11
	104O1	*SPSC*	12.28	0.00	7.94	2.21	0.00	0.00
	75F14	*SPSC*	3.99	0.00	0.42	1.80	0.00	0.29
	11OE1	*SPSD1*	3.71	0.47	6.07	1.11	0.00	0.31
	79G22	*SPSD2*	0.00	8.09	0.00	1.44	0.00	0.24
*S. spontaneum (*SES-208)	23E05	*SPSA*	23.83	2.61	0.46	1.58	0.00	0.08
	84H16	*SPSB*	3.70	1.79	6.25	1.01	0.05	0.09
	41F02	*SPSC*	7.75	0.00	3.44	1.87	0.00	0.00
	32E01	*SPSD1*	11.40	2.54	1.46	1.48	0.00	0.37
	69K24	*SPSD2*	25.54	0.00	1.93	0.91	0.00	0.34
	39L16	*SPSD2*	4.42	1.63	2.66	1.52	0.00	0.09

**Table 2:** Sequence similarity of *SPS* gene fragments between *Saccharum* and *Sorghum*

Sorghum					Saccharum					
Gene name	Chromosome position	Gene Length (bp)	cDNA Length (bp)	Exon	Gene name		Gene Length (bp)	Protein Length (aa)	Exon	Identity* (%)
*SbSPSA* (Sb09g028570)	Chr.9	12,456	3183	12	*SoSPSA* *SsSPSA*	*S. officinarum*	4658	468	9	99
						*S. spontaneum*	11,420	944	11	94
*SbSPSB* (Sb03g043900)	Chr.3	5575	3246	12	*SoSPSB* *SsSPSB*	*S. officinarum*	5849	1075	12	97
						*S. spontaneum*	5434	1107	12	91
*SbSPSC* (Sb05g007310)	Chr.5	5706	3216	9	*SoSPSC* *SsSPSC*	*S. officinarum*	6384	1062	9	91
						*S. spontaneum*	5452	1060	9	91
*SbSPSD1* (Sb10g025240)	Chr.10	9887	3030	14	*SoSPSD1* *SsSPSD1*	*S. officinarum*	10,881	982	13	100
						*S. spontaneum*	9532	567	12	99
*SbSPSD2* (Sb04g005720)	Chr.4	6689	2880	13	*SoSPSD2* *SsSPSD2*	*S.officinarum*	6989	931	13	98
						*S. spontaneum*	6885	956	13	98

### Homologs and allelic haplotype analysis of *SPS*

To identify *SPS* homologs and alleles in the selected BACs, analysis of conserved synteny was performed (Additional file 4). We observed five highly similar synteny blocks among *S. officinarum*, *S. spontaneum* and *Sorghum bicolor*, indicating that the two *Saccharum* species contained 5 *SPS* gene family members. Comparison within each synteny block across the three species showed high sequence identity at DNA level and conserved gene order (Additional file 4). For instance, three *SPSD1*-containing BAC contigs (LA110E11, SES32E01 and SES69 K24) were identified in *Saccharum*, two of which were allelic haplotypes from *S. spontaneum* and one from *S. officinarum*. Meanwhile, the orthologous region in *S. bicolor* was also displayed under the three BAC contigs. These sequences shared similar synteny blocks, suggesting that SES32E01 and SES69 K24 were allelic haplotypes in *S. spontaneum*. Similar results were observed in *SPSA*, *SPSB* and *SPSC* (Table 1). The *SPS* orthologs in the two species were highly similar, with sequence identities ranging from 95.6 to 100% under the pairwise comparison (Additional file 5).

**Fig. 1:**
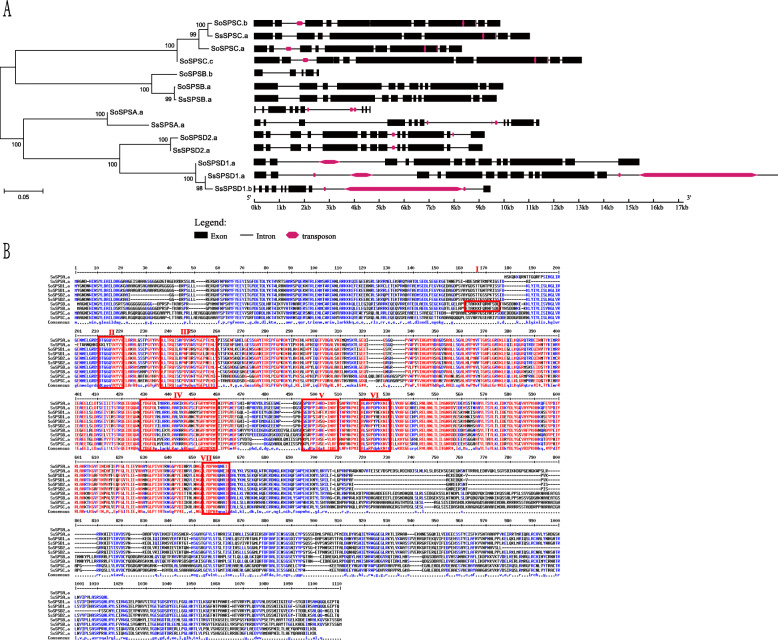
Gene structure (**a**) and multiple alignment analysis (**b**) of *SPS*. Ss and So indicate two *Saccharum* species, including *S. spontaneum* and *S. officinarum*, respectively. For Fig. 2b, regions of interest were masked with red rectangles: light regulated phosphoserine (I), putative F-6-P binding site (II), 14–3-3 regulated phosphoserine and UDP-G binding domain (III), the osmotically regulated phosphoserine (IV) and various aspartate-proline pairs (DP motif, V, VI, VII)

To further compare the *SPS* allelic haplotypes, we compared the exon-intron structures of the 14 *SPS* sequences in *S. officinarum* and *S. spontaneum* (Fig. 1a). The *SPS* genes were clustered together and the alleles are indicated with a, b or c. As expected, most orthologous/paralogous pairs showed similar exon-intron structure. For instance, three *SPSC* alleles (*SoSPSC.a*, *SoSPSC.b* and *SoSPSC.c*) were identified in *S. officinarum* and one allele (*SsSPSC.a*) was identified in *S. spontaneum*. Despite the overall similarity in gene structure, frequent divergence was also observed among the orthologs and haplotypes, though these proteins were highly conserved at amino acid level. We observed longer gene length and more exons (11 v.s. 9) in *SsSPSA.a* compared to *SoSPSA.a*. Notably, one exon was inserted after the second exon in *SsSPSA.a* and this gene appeared to possess an additional exon at the end of sequence. In addition, we identified three *SPSB* sequences (*SoSPSB.a*, *SoSPSB.b* and *SsSPSB.a*) in *S. officinarum* and *S. spontaneum*. *SoSPSB.a* and *SsSPSB.a* showed highly similar exon-intron structure, while the allelic haplotype *SoSPSB.b* was quite divergent compared to other *SPSBs*. *SoSPSB.b* was shorter in gene length and possessed fewer exons compared with *SoSPSB.a* and *SsSPSB.a*. This could be due to allelic variation or more likely resulted from incomplete genome assembly. Similar results were also observed in *SPSD1*. Exons in *SsSPSD1.b* tended to be shorter and fewer in number than in *SsSPSD1.a* and *SoSPSD1.a.*

Furthermore, we annotated the transposable elements (TEs) within the introns of the *SPS* genes (Fig. 1a). Four *SPS* members with the exception of *SPSB* were revealed to contain TE. TE insertions existed in the second intron of *SPSC* and the last intron of *SPSD2* in *S. officinarum* but were absent in *S. spontaneum*. In addition, a large TE was present in *SPSD1* from *S. spontaneum*, suggesting genomic expansion of *SPS* genes existed in *S. spontaneum*.

### Multiple alignment analysis

We performed a multiple alignment analysis for the *Saccharum SPS* genes and regions of interest were marked with red rectangles, including light-regulated phosphoserine, putative F-6-P binding site, 14–3-3 regulated phosphoserine and UDP-Glu binding domain, osmotically regulated phosphoserine and various aspartate-proline pairs (Fig. 1b). High sequence similarity was observed in the middle part of SPS proteins. As expected, the F-6-P binding sites and UDP-Glu binding domains are highly conserved at the amino acid level (Fig. 1b, II and III) in most sugarcane SPS proteins. Similarly, the osmotically regulated phosphoserine (IV) and various aspartate-proline pairs (V, VI and VII) are conserved as well, suggesting that these regions play important roles in sugar production. We also observed some mutations that may differentiate the functions of SPS proteins. For instance, a couple of mutations in the SsSPSA F-6-P binding domain (II) likely modified its F-6-P binding activity. A conversion from Serine (S) to Leucine (L) in SPSD family possibly influenced its function in UDP Glu binding (III). Remarkably, light regulated phosphoserines (I) were highly divergent, suggesting that the *SPS* genes played different roles in response to light regulation. We further investigated the *cis*-elements in the promoters of the 14 *SPS* genes (Additional file 6). *Cis*-elements that are related to the circadian clock, such as circadian and E-box, were observed in most of *Saccharum SPSs*. In addition, *cis*-elements involved in abiotic stress were predicted in the promoter region. For instance, ABREs (ABA-responsive elements) were identified in 6 *SPS* promoters and MYB-binding sites (MBSs) were found in 11 *SPS* promoters. These results suggested that *Saccharum SPS* genes might be regulated by the circadian clock and abiotic stress.

### Phylogenetic and evolutionary analysis of *SPS* genes in plants

**Fig. 2:**
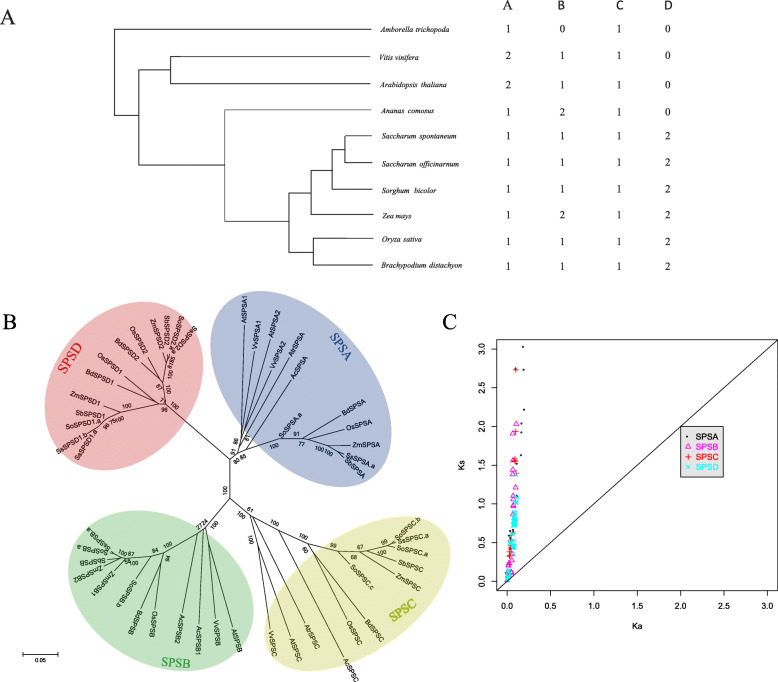
Evolutionary analysis of *SPS* family. **a** The distribution of *SPS* subfamilies in different plants. **b** Phylogenetic analysis of *SPS* gene family in different plants including *S. bicolor*, *S. officinarum*, *S. spontaneum*, *Zea mays*, *Oryza sativa*, *Arabidopsis thaliana*, *Vitis vinifera*, *Brachypodium distachyon*, *Ananas comosus* and *Amborella trichopoda*. **c** Ka/Ks values of the *SPS* subfamilies

To further investigate the evolutionary history of sugarcane *SPS* genes, we first analyzed *SPS* genes from two dicotyledonous plants (including *Arabidopsis thaliana* and *Vitis vinifera*), five monocotyledonous plants (including *Ananas comosus*, *S. bicolor*, *Brachypodium distachyon*, *Zea mays* and *Oryza sativa*) and a sole surviving sister species of all other living flowering plants (*Amborella trichopoda*) (Fig. 2a). All the *Saccharum SPS* genes identified in this study including alleles were included in the phylogenetic analysis (Fig. 2b). The result showed that selected plant *SPSs* were clustered into 4 classes (*SPSA, SPSB, SPSC* and *SPSD*). Similar to a previous study [4], *SPSA, SPSB* and *SPSC* sub-families are present in both monocotyledonous and dicotyledonous plants; while the *SPSD* gene family only exists in monocotyledonous plants. This result indicated that *SPSD* genes emerged more recently after the monocot-dicot divergence. In addition, the *SPSD* gene family had a closer phylogenetic relationship with *SPSA* than *SPSB* and *SPSC*. In *A. trichopoda*, we only identified two *SPS* genes (*SPSA* and *SPSC*) (Fig. 2a).

The non-synonymous to synonymous substitution rate (Ka/Ks) is an indication of selective pressures. A Ka/Ks ratio < 1 is consistent with a history of negative selection, while Ka/Ks ratio > 1 indicates a strong positive selection [26]. We performed a pairwise comparison within each *SPS* gene from selected plants (Fig. 2c). Almost all *SPS* genes showed that Ka/Ks values were lower than 1, suggesting that these family members were under strong purifying selection. To further identify the evolutionary forces acting on the sugarcane *SPS* genes after the divergence of *S. officinarum* and *S. spontaneum*, we investigated the Ka/Ks values of *SPS* genes in *S. bicolor*, *S. officinarum* and *S. spontaneum* (Additional file 7).

### Expression profiles of *SPS* genes in the different tissues of three developmental stages

To investigate the expression pattern of *SPS* gene families, two transcriptome databases (See materials and methods for details) were used for the expressional analysis from two *Saccharum* species (*S. spontaneum* and *S. officinarum*).

**Fig. 3:**
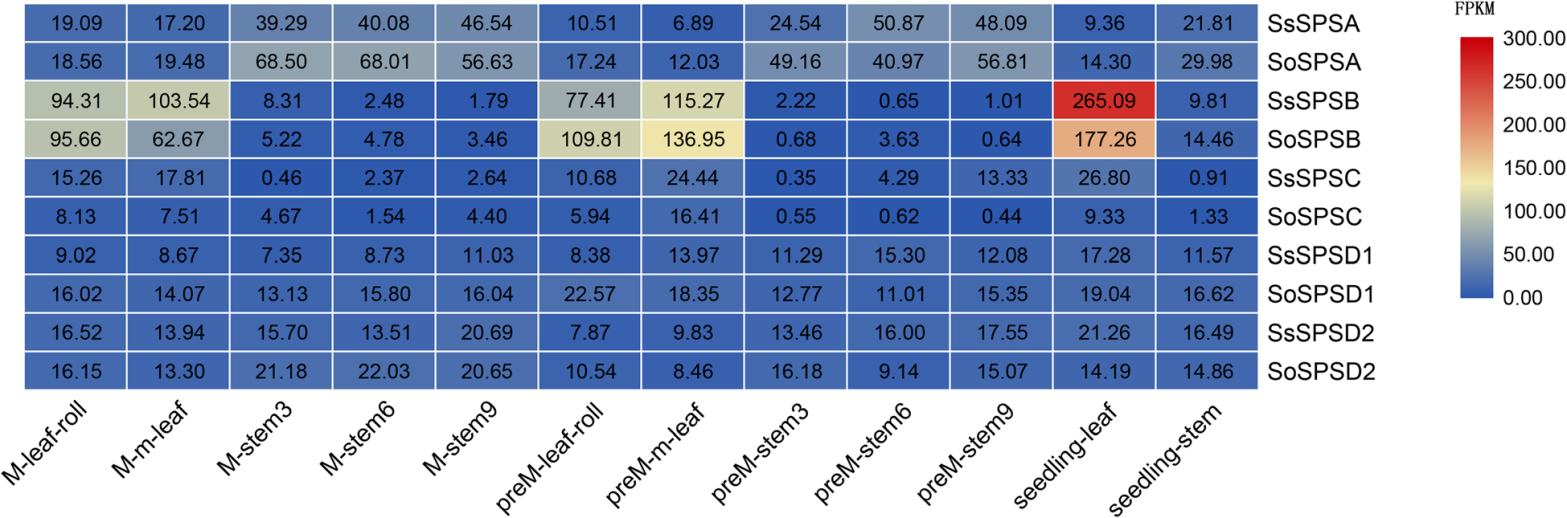
Expression profiles of *SPS* genes in various samples in two *Saccharum* species. Seedling, preM and M represent three developmental states: seedling, pre-mature and mature. In each stage, samples include leaves and internodes

The expression of *SPS* genes at three different developmental stages (seedling, pre-mature stage and mature stage) were clustered into two trends, demonstrating the significant expressional preference in the stem or leaves of the two species (Fig. 3). One trend is that the genes were much more highly expressed in leaves opposed to the stem, including *SPSB* and *SPSC* genes at these three developmental stages, which was consistent with a previous study [27]. Furthermore, *SPSB* expression was higher than *SPSC*, indicating that *SPSB* was the dominant gene expressed in the leaves and functioned in the green tissues of the two *Saccharum* species. The other trend is that the genes were expressed at significantly higher levels in the stem compared to those in leaves at all developmental stages, in particularly *SPSA, SPSD1* and *SPSD2* in *S. officinarum* and *S. spontaneum.* Similarly, *SPSA* was the major gene specifically expressed in the stem, especially at the mature stage, which is similar to a previous study that one sugarcane *SPS* gene was expressed in internodes [17]. Meanwhile, *SPSA* gene was the highest expressed of all *SPS *genes and higher in *S. officinarum* than in *S. spontaneum,* suggesting *SPSA* may play an important role in the transportation and storage of sugarcane sugar.

Interestingly, *SPSB* expression level was higher in the leaves of *S. officinarum* compared with *S. spontaneum* at the pre-mature stage, while more *SPSA* transcripts accumulated in the internodes of *S. officinarum* at the mature stage than *S. spontaneum* (Fig. 3), suggesting the differential expressions of *SPSA* and *SPSB* may contribute to the differences in sugar yields between *S. officinarum* and *S. spontaneum*.

### Expression profiles of *SPS* genes in the segments of developmental gradients in leaves

**Fig. 4:**
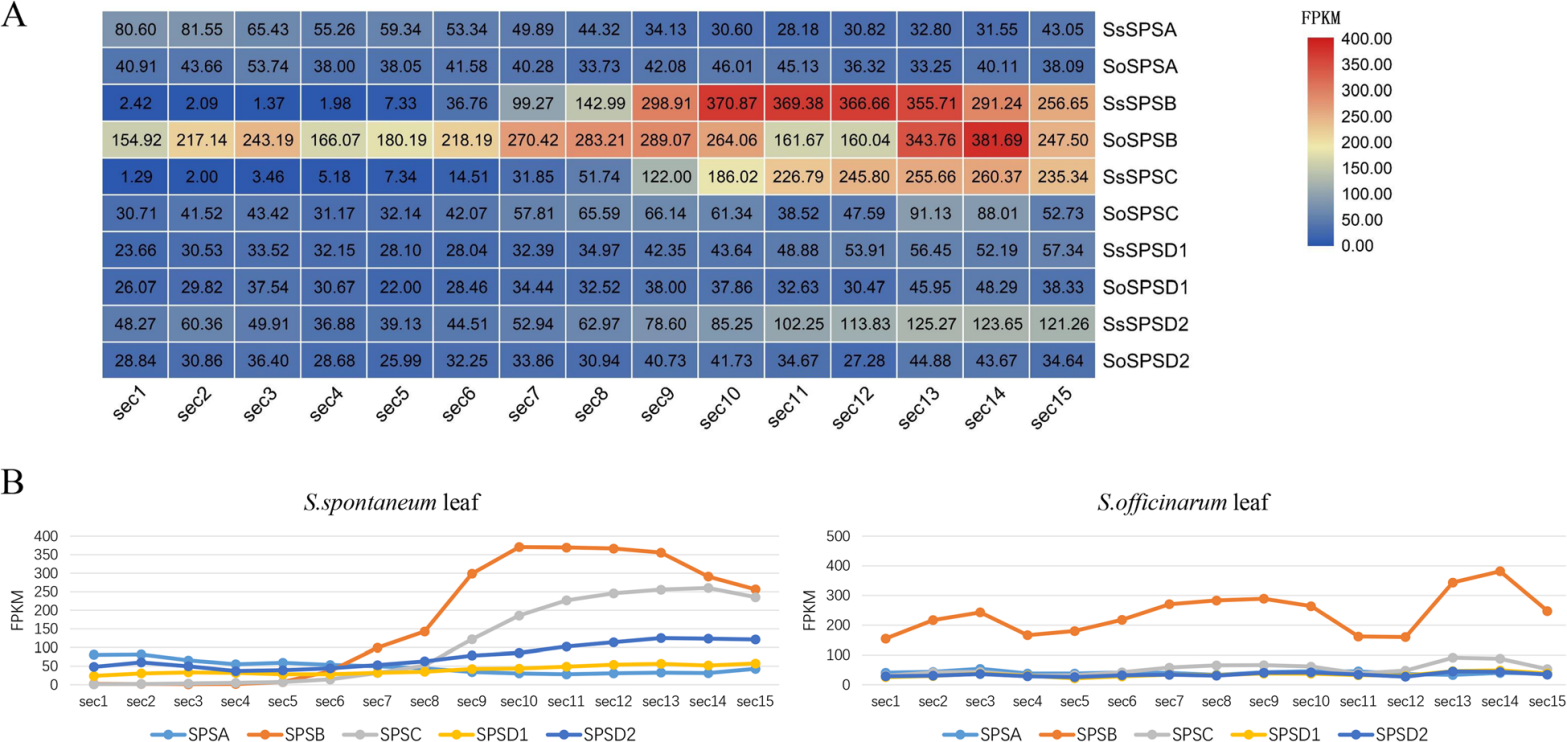
Expression profiles of *SPS* genes in gradient developmental leaves in two *Saccharum* species. Leaf samples in the seedling stage were divided into four gradient intervals (a basal zone, sink tissue; a transitional zone, going through the sink-source transition; a maturing zone and a mature zone, active C4 photosynthetic zones), including 15 segments (sec1 to sec15). **a** The heat map showing the expression levels of all SPS genes. s. **b** Comparative analysis of the trends in expression of the two species in gradient developmental leaves

Based on the above findings, the present study further analyzed the *SPS* expression patterns in the segments of development gradients in leaves and discovered that certain expressional patterns in almost all genes in these two species (Fig. 4a). We found that *SPS* gene expressions in *S. spontaneum* were consistent with the continuous developmental gradient in leaves, while there were three small peaks in *S. officinarum* (Fig. 4b). In additional, the higher specific expression of *SPSB* in the leaves of the two species were demonstrated (Fig. 4).

Interestingly, the *SPSC* expression level was lower than *SPSB* although the expression patterns were similar, indicating that the functions of *SPSB* and *SPSC* were complementary. Furthermore, this study also found that the *SPSA*, *SPSC* and *SPSD2* genes in *S. spontaneum* had higher than those in *S. officinarum,* relatively (Fig. 4a).

### SPS protein levels in the two *Saccharum* species

**Fig. 5:**
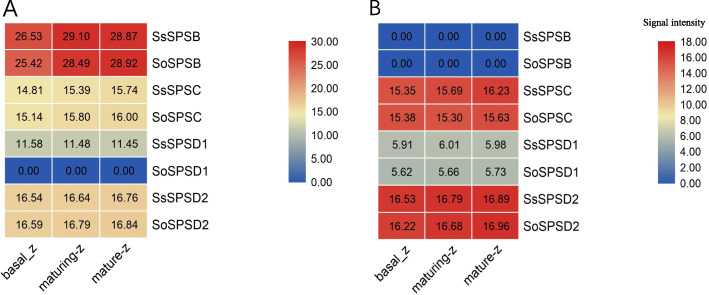
The relatively content of SPS protein in gradient developmental leaves (**a**) and stems (**b**) in two *Saccharum* species. The developmental leaves and stems were all divided into three developmental blocks: basal-zone (basal-z), maturing-zone (maturing-z), mature-zone (mature-zone)

To further explore the SPS protein levels in sugar synthesis source tissue (leaf) and sucrose accumulation sink tissue (stem), we performed proteomics analysis on a gradient in developing leaves and mature stem tissues (Fig. 5). In leaves, the relative protein content (log2 the intensity of protein) of SPSB was highest among the gene families, which was consistent with transcriptome data. The content of SPSD2 was much higher than that of SPSB, but only slightly higher than SPSC. The protein content of SPS only presented very limited variation between the two *Saccharum* species, except for SPSD1. The protein contents of the SPS gene families in stem were much different from those in leaf tissue, supporting the theory that SPSs are involved in the distribution of sugar. Importantly, in stem tissue SPSC and SPSD2 presented protein levels similar to leaf tissue, while, SPSB and SPSD1 were much lower than in leaf. These results suggested that the SPSB and SPSD1 contributed to the synthesis of sugar rather than SPSC and SPSD2. In addition, SPSA protein was undetectable in the examined tissues of these two *Saccharum* species, indicating the limited contribution of SPSA.

### Sugar contents in the two *Saccharum* species

To further analyze the SPS functions in sugarcane, metabolomics analysis was carried out and the changes in small molecule concentrations that were closely related to the phenotype of sucrose trait in two *Saccharum* species were examined. Based on the two sets of metabolome data from leaves at seedling and stems at mature stage, there were two metabolites, which were directly associated with SPS enzymes, F-6-P (a substrate during SPS catalyzing the synthesis of sucrose) and sucrose (end products of SPS catalytic pathway).

**Fig. 6:**
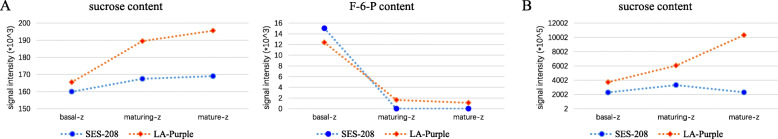
Metabolomics analysis in gradient developmental leaves (**a**) and stems (**b**) in two *Saccharum* species. The sampling methods are consistent with the proteomics analysis. The comparative analysis of the metabolites of sucrose and fructose-6-phosphatein in leaves and stem internodes of two species were performed, Fructose − 6-phosphate was not detected in the stems of either species

For the gradient of developing leaf, an increasing trend of sucrose content was found in *S. officinarum* and *S. spontaneum* as the leaves became into mature (Fig. 6a), while a generally decreasing trend of F-6-P content was found in the two species, indicating SPS performed the same functions during the sucrose synthesis in the two varieties. For the stems at different developmental states, sucrose content showed different tendencies among three species, such as a significant increase in *S. officinarum* and increasing from young stems to the maturing zone and then declining from the maturing zone to the mature zone in *S. spontaneum* (Fig. 6b).The levels of F-6-P were almost not detectable in three stem nodes from both *S. officinarum* and *S. spontaneum*. Whereas the sucrose contents in the seedling leaf and the stems were higher in *S. officinarum* than in *S. spontaneum.*

## Discussion

### Gene evolution of *SPS* family

In this study, we used an extensive collection of plant *SPS* gene members for phylogenetic analysis, and the results confirmed the previous classification [4], and revealed the existence of three groups (*SPSA/B/C*) containing both monocotyledonous and dicotyledonous and one Poaceae species specific *SPSD* (Fig. 1b). *A. trichopoda* only contained two *SPS* genes (*AtrSPSA* and *AtrSPSC*), suggesting that *SPSB* newly evolved after the divergence of *A. trichopoda* and other flowering plants (Fig. 2a). The evolutionary history of *Saccharum SPS*, which was sorted chronologically, from ancient to more recent, was *SPSA/SPSC, SPSB, SPSD1/SPSD2*.

Previous studies showed that *SPSD* genes emerged after the monocot-dicot divergence [28]. In our study, phylogenetic analysis more precisely showed that the *SPSD* genes likely evolved from the *SPSA* clade (Fig. 2b) and were clustered into two groups, *SPSD1* and *SPSD2*.Interestingly, *SPSD* genes only existed in Poaceae plants rather than in *A. comosus*, which was an out group to the Poaceae lineage in the *Poales* order [29], suggesting that *SPSD* genes evolved separately within the Poaceae clade. Within the coding regions, the Ka/Ks ratios of *SPSD* genes were much less than 1, indicating that purifying selection was the dominant force driving the evolution of *SPSD* genes after the speciation of the two *Saccharum* species. Molecular evolution analysis revealed that negative selection drove the evolution of these genes, suggesting their functional importance.

*Sorghum* is the closest relative in the diploid genera of *Saccharum*. Comparative analysis of the orthologs between *Sorghum* and *Saccharum* made it possible to investigate the specific evolutionary events after the polyploidization of *Saccharum*. Previous studies indicated that polyploidy was a powerful player in the acceleration of evolutionary adaptation [30]. One research group detected the rapid spread of beneficial mutations in tetraploid yeast in response to growth on a poor carbon source [31]. To investigate the evolutionary pressure of *SPS* genes in polyploidy *Saccharum*, we calculated the Ka/Ks ratios in *S. bicolor*, *S. officinarum* and *S. spontaneum* (Additional file 7). Almost all the pairwise comparisons of Ka/Ks that were statistically significant were lower than 1 (Fisher test, *p* < 0.05), indicating that *SPS* genes in *Sorghum* and *Saccharum* were undergoing purifying selection. However, the Ka/Ks ratios within *Saccharum* were significantly higher than between *Sorghum* and *Saccharum* (mean = 0.22 versus mean = 0.12, Mann-Whitney-Wilcoxon test, *p* = 0.01775). This result showed that polyploidy relaxed selection pressure of *SPS* genes in *Saccharum*, which is an expected outcome of gene redundancy in the polyploids.

### TEs influence the evolution of *SPS* genes

TE are a dominant feature of most flowering plant genomes and greatly contribute to genome evolution and diversity [32]. TEs are known to have a *cis*-effect on gene regulation since the likely DNA and histone methylation are often associated with the repetitive sequences. A previous study showed that high levels of TE variation was observed between *S. officinarum* and *S. spontaneum* [33]. In our research, comparison of *SPS* genes between the two sugarcane species displayed frequent TE variation, indicating that TEs could play important roles in *SPS* evolution before and after the divergence of *S. officinarum* and *S. spontaneum*. For instance, two adjacent TEs were inserted into the seventh exon of *SoSPSC.a*, *SoSPSC.b* and *SsSPSC.a*, suggesting that the events happened before the divergence of the two sugarcane species (Fig. 2a). However, another TE insertion event was only observed in the second intron of *SoSPSC.a* and *SoSPSC.b* rather than *SsSPSC.a*, suggesting that *SPSCs* were undergoing independent evolution after divergence of the two *Saccharum* species.

*SPSD* genes were unique in monocots and our results uncovered that they were likely undergoing quite different evolution fate. *SoSPSD2.a* and *SsSPSD2.a* were conserved in exon-intron order, gene length as well as TE distribution (Fig. 2a). However, it seemed that gene structure of *SPSD1* was less conserved and the last exon of *SsSPSD1.a* was interrupted by a large TE insertion. Moreover, the *SsSPSD1.b* displayed a quite different exon-intron structure as well as a large TE variation compared to its allelic haplotype *SsSPSD1.a.* These results demonstrated that the two copies of newly evolved *SPS* subfamily tended to be functional diverse in sugarcane and possibly TE variation was responsible for the diversity, providing the first evidence that TE contributed to gene evolution in *Saccharum*.

### Functional divergence of *SPS* genes between *S. officinarum* and *S. spontaneum*

RNA-seq analysis revealed that the expression of *SPSB* in *S. officinarum* leaves were higher than that in *S. spontaneum* leaves at the pre-mature stage (Fig. 3). Furthermore, the expression of *SPS* in leaves displayed consistent trend between these two *Saccharum* species as a developmental gradient progressed (Fig. 4), indicating that the expression of *SPS* was responded to photosynthesis in the seedling leaf.

*SPSB* presented consistently high expression in *S. officinarum* which was consistent with the highest relative content of SPSB protein based on proteomics analysis, indicating *SPSB* may be the major gene of sucrose synthesis in leaf. Combined with metabolome data, we found in leaves at the seedling stage that sucrose increases and F-6-P decreases, which was in line with the trend of expression of *SPS* (especially the *SPSB* gene with high expression level) mentioned above which catalyzes the synthesis of sucrose using substrate of F-6-P. Therefore, in the source tissue leaves, we speculate that SPS genes play a key role in sucrose synthesis which is linked with the development of leaves in these two *Saccharum* species. Further correlation analysis revealed there were significant overall relationships between the expression of *SPSB* and the content of sucrose (positive, r^2^ = 0.97 or 0.93) or F-6-P (negative, r^2^ = 0.84 or 0.90) both in *S. officinarum* and in *S. spontaneum* (Additional file 8). Since the characteristics of high and low sugar in cultivated sugarcane were respectively contributed by *S. officinarum* and *S. spontaneum,* the gene expression of *SPSB* may play a significant role in sucrose synthesis and accumulation. These results revealed that *SPSB* played a vital role in sugarcane leaves and they might be responsible for the difference of sugar at the production process of growth. However, considering the limited data points for correlation analysis, further verification of experimental data is required to support this speculation.

Besides *SPSB*, *SPSA* also showed different expression patterns at seedling and mature stages between the two species (Fig. 3). *SPSA* was expressed at higher levels in *S. officinarum* stems than in *S. spontaneum* stems, which may be caused by different gene structures given that there are 9 exons in *SoSPSA* and 11 exons in *SsSPSA* (Fig. 1a). Interestingly, when the expression of *SPSA* in different segments of the developing stem and metabolites were compared, a significant nonlinear negative relationship did exist among three species, except for one special case in *S. spontaneum* in which the sucrose content was to some extent correlated with the expression of *SPSA* (r^2^ = 0.84 or 0.90) in the stem (Additional file 8). This indicates that there could be other genes regulating sucrose synthesis in stem tissue or other influencing factors, resulting in differences in sucrose content in stems of the three species. Considering the lower expression of *SPS* genes in stem tissue than in leaf tissue and the higher expression levels of *SPS* genes in *S. officinarum* than in *S. spontaneum,* lower levels of sucrose synthesis in stems and high sucrose content in stem were mainly obtained through sugar transport rather than sugar synthesis.

The results revealed that *SPSD* duplicated within the Poaceae lineage. The gene expression data revealed that the two *SPSDs* had similar expression patterns, suggesting functional similarity in this gene subfamily. Furthermore, *SPSD* maintained similar lower expression levels in all tissues of sugarcane and there are no specific expressive features, which is different from wheat *SPS* genes but consistent with the research in sugarcane [34]. However, both *SPSD* gene copies were under strong purifying selection shown by their Ka/Ks ratio < 0.4 (Additional file 7), which demonstrated that the two *SPSDs* were not functionally redundant in *Saccharum*. Therefore, we assumed that two *SPSD* subfamilies shared similar gene functions while possessing divergent key functional roles in *Saccharum*. Proteome data showed that the protein content of SPSD2 was highest in the developing stem and SPSA protein was not detected in leaves and stem internodes. Combined with the recent replication events in which *SPSD* evolved from *SPSA* and the high levels of *SPSA* gene expression in the stem, we suspected that the high *SPSA* expression levels in stem tissue regulates the translation of the highly homologous genes of *SPSD,* which lead to the high protein levels of SPSD2 and free protein content of SPSA in stem tissue in both two species.

Interestingly, the present study found *SPS* expression was affected by photosynthesis and regulated by circadian rhythm. All SPS in the gradient of developing leaves displayed differing expression patterns, indicating *SPS* expression was synchronized with light assimilation in a species-specific manner (Fig. 4). Therefore, we assumed that the SPS contributed to the differential sugar accumulation between the two *Saccharum* species. Notably, SPS expression was sometimes regulated by other enzymes, for example, SPS had potential functions and contributed to sucrose synthesis when soluble acid invertase (SAI) was low [35].

Meanwhile, based on the high spatiotemporal specificity of *SPSB* and *SPSA* in the *SPS* gene family and the simultaneous comparative analysis of transcriptomes, metabolomes and proteomes, we suspect that the regulation of *SPSB* and *SPSA* gene expression are an important point in the formation of regulatory networks in sucrose traits among different species of *Saccharum.* Furthermore, sucrose accumulation is a complex process requiring the involvement of multiple genes for sugar synthesis, transport and the targeting regulation. In this way, different patterns of expression of *SPS* genes may be formed among different sugarcane species, leading to different levels of photosynthetic carbon fixation and even different sugar traits.

## Conclusions

We presented a comprehensive analysis of *SPS* genes in two *Saccharum* species, including *S. spontaneum* and *S. officinarum*. By analyzing and comparing these *SPS* genes, we concluded three major findings. Firstly, frequent structural variations and mutations were observed in *SPS* homologs as well as allelic haplotypes. In addition, comparison of *SPS* genes between the two *Saccharum* species displayed several TE insertion/deletion events, indicating that TEs might play important roles in *SPS* evolution before and after the divergence of the two species. Secondly, the *SPSD* subfamily in *Saccharum* was newly evolved from *SPSA* after the generation of Poaceae species. Molecular evolution analysis showed that all the *SPS* genes were under negative selection and selection pressure was reduced under the process of polyploidy. Thirdly, RNA-seq, metabolome and proteome data uncovered the different expression patterns of the *SPS* gene family between *S. officinarum* and *S. spontaneum*, suggesting that *SPSA* and *SPSB* are possibly responsible for the fundamental differences in sugar yields at different stages. The high expression of *SPSB* in developing leaves may play a direct and key role in sucrose synthesis at the source organization, and the accumulation of high sucrose levels In the stem there may be a more complex regulatory network in which *SPSA* and *SPSD* genes are indirectly involved.

## Methods

### Plant materials and RNA extraction

Sugarcane species LA-Purple [36, 37] (*S. officinarum*, 2n = 8x = 80, originated in USA) and SES208 [38] (*S. spontaneum*, 2n = 8x = 64, originated in USA) [39, 40] that were deposited in the National Germplasm Repository of Sugarcane (Yunnan, China) and used in the present study. Plants were grown in plastic pots under greenhouse conditions (14:10 L/D, 30 °C L/22 °C D and 60% relative humidity) and standard growing practices in the sugarcane experiment field at Fujian Agricultural and Forestry University (Fuzhou, China). For the investigation of the different developmental stages and the gradient developmental leaf analysis experiments were performed as previously described [41]. Tissue of developmental leaf was collected 3 h into the L period as detailed by Li et al. [42] and the collection of other tissues was detailed by Ming et al. [29]

RNA from various sugarcane tissue samples were extracted using Trizol (Invitrogen, USA) according to the manufacturer’s protocol. RNA was digested with DNase I (NEB, USA) and the RNA integrity was assessed using the Agilent Bio-analyzer 2100 system (Agilent Technologies, CA, USA).

### BAC libraries

A list of BAC libraries were constructed from the haploid genome of *S. spontaneum* (AP85–441, 2n = 4x = 32) and *S. officinarum* (LA Purple, 2n = 80). Nuclei were isolated from young leaf tissues following the protocol in [43]. Briefly, high molecular weight DNA was extracted and digested into fragments with *HindIII*. Approximately 100 kb fragments were isolated and inserted into the pSMART BAC vector (Lucigene, LA). BAC clones which contained potential *SPS* genes were sequenced using an Illumina Hiseq 2500. There were 74,880 clones in BAC library of LA Purple based on 195–384 well plates with 150 kb of an average insert size, which provided the 1.5 x and 12 x coverage of the octoploid genome and the monoploid genome, respectively. For AP85–441, the BAC library was consisted of 38,400 clones (in the 100–384 well plates with an average insert size at 120 kb), which was an about 1.5 x coverage of the haploid (tetraploid) genome and an about 6 x coverage of the monoploid genome.

### Assembly, annotation of BAC reads and identification of *SPS* gene family

The raw reads were assembled using SPAdes 3.6.2 with default parameters [44]. Firstly, we designed the probes using *Sorghum SPS* sequences (Additional file 9) and matched the probes against sugarcane BAC libraries. We then performed De novo assembly and gene annotation for the selected BACs*.* To get a better annotation, TEs and tandem repeat sequences were identified. The assembled contigs were screened using RepeatMasker [45] for TE annotation and to the DNA subway system (http://dnasubway.iplantcollaborative.org/) for coding region annotation using the model of “Annotate a genomic sequence” referring to the tips on the web page (https://github.com/CyVerse-learning-materials/dnasubway_guide). For TE identification, annotation was confirmed if there was at least 60% identity and a minimum length of 50 bp with previously annotated plant TEs (ftp://ftp.plantbiology.msu.edu/pub/data/TIGR_Plant_Repeats/).The corresponding coding sequences were translated into protein by the EXPASy-translate tool (http://web.expasy.org/translate/).

*SPS* homologs from other plants, such as Arabidopsis, maize and so on, were downloaded from NCBI (http://www.ncbi.nlm.nih.gov/). A blast-based search was performed to identify SPS gene family from *S. officinarum* and *S. spontaneum*, as well as selected plants listed in Additional file 5. The cutoff was set as E-value less than 10^− 20^ and identity higher than 85%.

### Gene structure, phylogenetic and evolutionary analysis

The exon-intron structures were extracted from gene annotation data generated by DNA subway and further displayed in GSDS (Gene Structure Display Server) [46]. In addition, MultAlin [47] program was used to illustrate the multiple sequence alignment of amino acids and domains of interest for sugarcane SPS genes. Plant CARE (http://bioinformatics.psb.ugent.be/webtools/plantcare/html/) was used to predict cis-elements in *SPS* promoters.

To generate a phylogenetic tree, the predicted protein sequences from seven plants, including two *Saccharum* species (*S. officinarum* and *S. spontaneum*), *Zea mays*, *Sorghum bicolor*, *Arabidopsis thaliana*, *Brachypodium distachyon*, *Vitis vinifera*, *Ananas comosus, Oryza sativa* and *Amborella trichopoda* were initially aligned based on Clustal W2.0 [48]. Then we reconstructed the phylogenetic tree of *SPS* genes by the Neighbor-Joining method estimated by the JTT amino acid matrix implemented using the program MEGA 4.0 [49]. The pairwise deletion option was set in the NJ tree reconstruction and the accuracy of the tree topology was assessed by bootstrap analysis with 1000 resampling replicates.

Genes of *SPSA, SPSB, SPSC* and *SPSD* were initially aligned using Clustal W2.0 [48]. The aligned sequences were subject to KaKs_Calculator [50] to calculate the Ka and Ks values.

### Expression analysis using RNA-seq data

For two blocks of expression profile, the first batch of samples were from different tissues at three different developmental stages (seedling, pre-mature stage and mature stage), including 2 leaves (mature leaf and leaf roll) and 3 stalks (mature, maturing and immature stalk). In additional, the second batch of samples were segments of the gradient developmental leaf, which was divided into 15 clips in four zones: a basal zone (base, sink tissue), a transitional zone (going through the sink-source transition), a maturing zone and a mature zone (active C_4_ photosynthetic zones, fully differentiated).

Paired-end sequencing (100 bp reads length) was performed using the HiSeq 2500 platform. The raw data was obtained from a list of RNA-seq libraries and initially filtered using Trimommatic [51] with default parameters. Then Tophat program was performed to map clean reads to assemble BAC contigs and the FPKM values for each SPS gene were calculated using cufflinks [52].

### Metabolomics experiments

Leaves in seedling and stems in the mature stage were sampled from two species, LA-Purple (*S. officinarum*), SES 208 (*S. spontaneum*), respectively. For the leaf samples, we selected three segments during different developmental stages, 0–2 segments (basal zone), 7–9 segments (maturing zone) and 13–15 segments (mature zone). For stem samples, the internodes selected were the same as in the experiments involving RNA-seq, three zones (basal zone, maturing zone, mature zone), stem 3, 6, 9 in *S. spontaneum* and stem 3,9,15 in *S. officinarum*. Samples were harvested and ground with a ball mill precooled with liquid nitrogen, brought back to extract and derivatize the metabolites. Chromatographic analyses of derivatized samples were achievedusing a random injection sequence, on an Agilent 7890B GC (Agilent, Atlanta, GA, USA), coupled to a Pegasus HT time-of-flight mass spectrometer (LECO, St. Joseph, MI, USA), equipped with a Gerstel MultiPurpose Sampler (MPS) (Gerstel, Mülheim an der Ruhr, Germany). Five experimental replicates of each tissue were tested. Raw data was processed using ChromaTOF (version 4.51.6, LECO, St. Joseph, MI, USA) to identify the metabolites. Signal redundancy per metabolite was manually corrected and based on the normalized mean area of selected ions, quantitative measurements of analytes were performed after normalization using the IS. Next, by matching the mass spectra with those of authentic standards, public and commercial databases (NIST, Fiehn and Golm metabolome databases), the metabolites were identified. Chemical standards were purchased from Sigma (St. Louis, MO, USA) or Fluka (Milwaukee, WI, USA). For comparing the abundances of metabolites, the data matrix consisting of mass features and peak area values were exported from ChromaTOF to Excel. The mean peak area abundance values from five technical replicates were calculated after normalization to IS [53].

### Proteomics experiments

To investigate the proteomic changes among two sugarcane species, the leave and stem samples analyzed were of the same stage as those used for the metabolomics experiments. To prepare the protein, one gram of fresh tissues was collected and the experimental method was referred to the FASP procedure [54] and the manufacturer’s instructions (Thermo Fisher Scientific) following the TMT labeling procedure. We then prepared the sample to be fractionated by the strong cation exchange (SCX) chromatography as previously described [55]. On a nanoflow HPLC (Proxeon Biosystems, now Thermo Fisher Scientific), we performed RP-HPLC separation. The resolution for MS/MS spectra was set to 17,500 at m/z 200 and the normalized collision energy was 29%. Reporter ion quantitation was based on the extraction of the TMT reporter ion signals of each peptide by MaxQuant software. The contents of proteins with the value of peak area were then quantified by summing reporter ion counts across all peptide matches, and then normalized by assuming equal protein loading across all samples.

## Supplementary information

**Additional file 1.** Gene annotation of BAC assembly.

**Additional file 2. **Gene identification of *SPS* genes in the *S. spontaneum* genome by Blastp with the sequence of *SPS* genes from BAC libraries. Two genes (*Sspon.007C0001731* and *Sspon.004A0021251*) were reannotated.

**Additional file 3. **The protein sequences of SPS genes in *S. spontaneum* genome.

**Additional file 4. **Syntenic comparison of genomic DNA containing *SPS* genes among *S. officinarum*, *S. spontaneum* and *S.bicolor*. Homologs are indicated by arrows with same color. The asterisks indicate single-copied genes in the selected gene set.

**Additional file 5. **Pairwise comparison of *SP*S homologs in *Saccharum*.

**Additional file 6. ***Cis*-elements in *SPS* promoters.

**Additional file 7. **Ka, Ks comparison of *SPS* orthologs/alleles in *S.bicolor*, *S. officinarum* and *S. spontaneum.*

**Additional file 8. **Correlation analysis between the expression of *SPS* genes and content of the metabolites in gradient developmental leaves (A, left) and stems (B, right) in *Saccharum*. The correlation coefficient (R^2^) and dependent equation of each groups data is marked near the correlation curve. R^2^ > 0.9 was considered to be significantly correlated.

**Additional file 9. **The information for probes used to identify *SPS*-containing BACs.

## Data Availability

The 14 assembled BAC contigs were submitted to NCBI with the accessions KU685404-KU685417. Phylogenetic data (the alignments and phylogenetic trees) have been deposited to TreeBase and are accessible via the URL: http://purl.org/phylo/treebase/phylows/study/TB2:S19156.

## Abbreviations

ABREs: ABA-responsive elements
Developmental blocks: Basal-zone (basal-z), maturing-zone (maturing-z), mature-zone (mature-z)
Developmental states: Seedling (seedling), pre-mature (pre-M), mature (M)
F-6-P: Fructose-6-Phosphate
GS: Glutamine synthetase
GSDS: Gene Structure Display Server program
LTR: Long Terminal Repeat
MBSs: MYB-binding sites
Ka/Ks: Non-synonymous to synonymous substitution rate
ORFs: Open reading frames
SPS: Sucrose phosphate synthase
S-6-P: Sucrose-6-Phosphate
SPP: Sucrose phosphate phosphatase
Ss: *Saccharum officinarum, S. officinarum*
So: *Saccharum spontaneum, S. spontaneum*
SAI: Soluble acid invertase
TEs: Transposable Elements
UDP-G: UDP-Glucose
