# Supplementary material for: Comparative analysis of sucrose phosphate synthase (SPS) gene family between Saccharum officinarum and Saccharum spontaneum
Source: BMC Plant Biol. 2020 Sep 14;20:422. doi: 10.1186/s12870-020-02599-7 (PMC7488781; doi:10.1186/s12870-020-02599-7)
Supplement: Supplementary file 1 — Additional file 1. Gene annotation of BAC assembly. [file 12870_2020_2599_MOESM1_ESM.doc]

**Additional file 1.** Gene annotation of BAC assembly.

| **GeneID** | **Protein Length** | **Gene Length** | **BACid** | **Best blast hit** | **Identity** | **E-value** | **Description** |
| --- | --- | --- | --- | --- | --- | --- | --- |
| **SuBAC0000010** | 82 | 342 | LA104O01 | XP_002444537 | 61.667 | 1.26E-14 | hypothetical protein SORBIDRAFT_07g023490 [Sorghum bicolor]. |
| **SuBAC0000020** | 531 | 1851 | LA104O01 | XP_006650506 | 46.405 | 4.79E-30 | PREDICTED: phytochrome A-like [Oryza brachyantha]. |
| **SuBAC0000030** | 1067 | 5997 | LA104O01 | XP_002449293 | 90.825 | 0 | hypothetical protein SORBIDRAFT_05g007310 [Sorghum bicolor]. |
| **SuBAC0000040** | 98 | 294 | LA104O01 | AGT16872 | 55.556 | 2.31E-27 | Protein of unknown function DUF295 [Saccharum hybrid cultivar R570]. |
| **SuBAC0000050** | 477 | 7696 | LA110E11 | AGT15919 | 99.79 | 0 | purple acid phosphatase precursor [Saccharum hybrid cultivar R570]. |
| **SuBAC0000060** | 982 | 10881 | LA110E11 | AGT17001 | 99.491 | 0 | sucrose-phosphate synthase [Saccharum hybrid cultivar R570]. |
| **SuBAC0000070** | 372 | 3293 | LA110E11 | AGT15921 | 100 | 0 | hypothetical protein SHCRBa_179_G22_F_140 [Saccharum hybrid cultivar R570]. |
| **SuBAC0000080** | 380 | 4979 | LA110E11 | AGT16222 | 94.192 | 0 | formamidopyrimidine-DNA glycosylase-like protein [Saccharum hybrid cultivar R570]. |
| **SuBAC0000090** | 262 | 808 | LA110E11 | XP_004965761 | 48.344 | 1.23E-65 | PREDICTED: 36.4 kDa proline-rich protein-like [Setaria italica]. |
| **SuBAC0000100** | 85 | 918 | LA110E11 | AFK41124 | 70 | 3.88E-18 | unknown [Lotus japonicus]. |
| **SuBAC0000110** | 99 | 3622 | LA154P24 | NP_001148325 | 98.958 | 2.65E-63 | ubiquitin-like protein SMT3 [Zea mays]. |
| **SuBAC0000120** | 233 | 1649 | LA154P24 | XP_008656940 | 88.793 | 1.07E-144 | PREDICTED: uncharacterized protein LOC103636358 [Zea mays]. |
| **SuBAC0000130** | 593 | 4807 | LA154P24 | XP_002456840 | 94.376 | 0 | hypothetical protein SORBIDRAFT_03g043876, partial [Sorghum bicolor]. |
| **SuBAC0000140** | 316 | 1723 | LA154P24 | XP_002438527 | 69.231 | 7.46E-170 | hypothetical protein SORBIDRAFT_10g021570 [Sorghum bicolor]. |
| **SuBAC0000150** | 522 | 2444 | LA154P24 | AFW84172 | 91.031 | 0 | hypothetical protein ZEAMMB73_394344 [Zea mays]. |
| **SuBAC0000160** | 76 | 228 | LA154P24 | N.A. |  |  |  |
| **SuBAC0000170** | 516 | 8903 | LA154P24 | ABS89149 | 95.431 | 3.63E-107 | MATE [Sorghum bicolor]. |
| **SuBAC0000180** | 1075 | 5849 | LA154P24 | AEO46461 | 99.441 | 0 | sucrose phosphate synthase B [Saccharum hybrid cultivar ROC22]. |
| **SuBAC0000190** | 931 | 6989 | LA179G22 | ADM63847 | 95.539 | 0 | sucrose phosphate synthase [Saccharum hybrid cultivar ROC22]. |
| **SuBAC0000200** | 212 | 3088 | LA33C13 | CDM81076 | 50.909 | 1.58E-66 | unnamed protein product [Triticum aestivum]. |
| **SuBAC0000210** | 1062 | 6384 | LA33C13 | XP_002449293 | 90.715 | 0 | hypothetical protein SORBIDRAFT_05g007310 [Sorghum bicolor]. |
| **SuBAC0000220** | 425 | 1275 | LA33C13 | XP_002449294 | 84.954 | 0 | hypothetical protein SORBIDRAFT_05g007320 [Sorghum bicolor]. |
| **SuBAC0000230** | 77 | 231 | LA33C13 | XP_002449300 | 49.383 | 1.31E-15 | hypothetical protein SORBIDRAFT_05g007380 [Sorghum bicolor]. |
| **SuBAC0000240** | 98 | 294 | LA33C13 | AGT16872 | 57.407 | 5.02E-29 | Protein of unknown function DUF295 [Saccharum hybrid cultivar R570]. |
| **SuBAC0000250** | 440 | 1320 | LA33C13 | XP_002449301 | 83.669 | 0 | hypothetical protein SORBIDRAFT_05g007390 [Sorghum bicolor]. |
| **SuBAC0000260** | 332 | 6714 | LA33C13 | ACG35871 | 83.626 | 0 | glycerophosphodiester phosphodiesterase [Zea mays]. |
| **SuBAC0000270** | 396 | 5509 | LA34B02 | XP_002458993 | 97.985 | 0 | hypothetical protein SORBIDRAFT_03g043970 [Sorghum bicolor]. |
| **SuBAC0000280** | 370 | 4811 | LA34B02 | AFK32343 | 88.432 | 0 | putative auxin efflux carrier PIN5a [Zea mays]. |
| **SuBAC0000290** | 381 | 3302 | LA34B02 | ACG27604 | 92.632 | 0 | hydrolase-like protein [Zea mays]. |
| **SuBAC0000300** | 210 | 995 | LA34B02 | XP_002455971 | 34.672 | 6.42E-29 | hypothetical protein SORBIDRAFT_03g028280 [Sorghum bicolor]. |
| **SuBAC0000310** | 410 | 3587 | LA34B02 | XP_002456843 | 92.665 | 0 | hypothetical protein SORBIDRAFT_03g043920 [Sorghum bicolor]. |
| **SuBAC0000320** | 243 | 2294 | LA34B02 | XP_002456842 | 93.802 | 1.66E-153 | hypothetical protein SORBIDRAFT_03g043910 [Sorghum bicolor]. |
| **SuBAC0000330** | 550 | 5446 | LA75F14 | XP_002449293 | 89.636 | 0 | hypothetical protein SORBIDRAFT_05g007310 [Sorghum bicolor]. |
| **SuBAC0000340** | 468 | 4658 | LA84F06 | ADL70859 | 99.558 | 0 | sucrose phosphate synthase A [Saccharum hybrid cultivar ROC22]. |
| **SuBAC0000350** | 80 | 706 | LA84F06 | NP_001145960 | 64.103 | 2.24E-05 | uncharacterized protein LOC100279486 [Zea mays]. |
| **SuBAC0000360** | 122 | 515 | LA84F06 | AFW58210 | 42.391 | 1.70E-09 | hypothetical protein ZEAMMB73_655484 [Zea mays]. |
| **SuBAC0000370** | 114 | 358 | LA84F06 | XP_002442739 | 36.634 | 2.52E-10 | hypothetical protein SORBIDRAFT_08g002035, partial [Sorghum bicolor]. |
| **SuBAC0000380** | 173 | 2143 | LA84F06 | NP_001141281 | 87.931 | 5.46E-91 | uncharacterized protein LOC100273370 [Zea mays]. |
| **SuBAC0000390** | 179 | 1970 | LA84F06 | XP_002440257 | 100 | 1.09E-129 | hypothetical protein SORBIDRAFT_09g028590 [Sorghum bicolor]. |
| **SuBAC0000400** | 913 | 4078 | SES23E05 | XP_004961135 | 35.652 | 2.53E-05 | PREDICTED: protein CHUP1, chloroplastic [Setaria italica]. |
| **SuBAC0000410** | 284 | 852 | SES23E05 | XP_008657508 | 79.861 | 1.04E-134 | PREDICTED: ethylene-responsive transcription factor ERF094-like [Zea mays]. |
| **SuBAC0000420** | 944 | 11420 | SES23E05 | ADL70859 | 97.007 | 0 | sucrose phosphate synthase A [Saccharum hybrid cultivar ROC22]. |
| **SuBAC0000430** | 125 | 375 | SES23E05 | AFW58210 | 36.496 | 9.18E-08 | hypothetical protein ZEAMMB73_655484 [Zea mays]. |
| **SuBAC0000440** | 111 | 609 | SES23E05 | N.A. |  |  |  |
| **SuBAC0000450** | 178 | 4688 | SES23E05 | XP_002468143 | 34.389 | 4.19E-34 | hypothetical protein SORBIDRAFT_01g040370 [Sorghum bicolor]. |
| **SuBAC0000460** | 326 | 4514 | SES23E05 | XP_004961208 | 87.611 | 0 | PREDICTED: 60S ribosomal protein L18a-like [Setaria italica]. |
| **SuBAC0000470** | 250 | 2080 | SES23E05 | XP_002440258 | 95.359 | 7.53E-165 | hypothetical protein SORBIDRAFT_09g028600 [Sorghum bicolor]. |
| **SuBAC0000480** | 452 | 7374 | SES23E05 | XP_002441523 | 67.287 | 1.34E-162 | hypothetical protein SORBIDRAFT_09g028610 [Sorghum bicolor]. |
| **SuBAC0000490** | 277 | 3051 | SES23E05 | XP_008654509 | 97.122 | 0 | PREDICTED: uncharacterized protein LOC100217046 isoform X1 [Zea mays]. |
| **SuBAC0000500** | 281 | 3913 | SES23E05 | NP_001130475 | 87 | 3.26E-172 | uncharacterized protein LOC100191573 [Zea mays]. |
| **SuBAC0000510** | 317 | 951 | SES23E05 | XP_002441524 | 92.722 | 0 | hypothetical protein SORBIDRAFT_09g028640 [Sorghum bicolor]. |
| **SuBAC0000520** | 134 | 402 | SES23E05 | XP_008656041 | 85.075 | 1.21E-48 | PREDICTED: uncharacterized protein LOC103635339 [Zea mays]. |
| **SuBAC0000530** | 397 | 5043 | SES32E01 | AGT17003 | 98.737 | 0 | hypothetical protein SHCRBa_063_N24_F_320 [Saccharum hybrid cultivar R570]. |
| **SuBAC0000540** | 368 | 3090 | SES32E01 | AGT15921 | 98.652 | 0 | hypothetical protein SHCRBa_179_G22_F_140 [Saccharum hybrid cultivar R570]. |
| **SuBAC0000550** | 1040 | 17248 | SES32E01 | XP_002438738 | 93.558 | 0 | hypothetical protein SORBIDRAFT_10g025240 [Sorghum bicolor]. |
| **SuBAC0000560** | 180 | 3963 | SES32E01 | EMS51666 | 64.43 | 5.45E-52 | Auxin response factor 17 [Triticum urartu]. |
| **SuBAC0000570** | 477 | 5971 | SES32E01 | AGT16225 | 99.37 | 0 | purple acid phosphatase precursor [Saccharum hybrid cultivar R570]. |
| **SuBAC0000580** | 185 | 4266 | SES32E01 | AGT16999 | 87.981 | 3.02E-125 | hypothetical protein SHCRBa_063_N24_R_210 [Saccharum hybrid cultivar R570]. |
| **SuBAC0000590** | 729 | 4600 | SES32E01 | AGT15917 | 94.293 | 0 | vacuolar H+-pyrophosphatase [Saccharum hybrid cultivar R570]. |
| **SuBAC0000600** | 63 | 778 | SES39L16 | N.A. |  |  |  |
| **SuBAC0000610** | 109 | 3231 | SES39L16 | ACG46293 | 81.25 | 1.55E-26 | hypothetical protein [Zea mays]. |
| **SuBAC0000620** | 767 | 6405 | SES39L16 | XP_002453419 | 99.347 | 0 | hypothetical protein SORBIDRAFT_04g005710 [Sorghum bicolor]. |
| **SuBAC0000630** | 965 | 6885 | SES39L16 | ADM63847 | 99.896 | 0 | sucrose phosphate synthase [Saccharum hybrid cultivar ROC22]. |
| **SuBAC0000640** | 278 | 4231 | SES39L16 | XP_002451676 | 96.029 | 1.44E-172 | hypothetical protein SORBIDRAFT_04g005730 [Sorghum bicolor]. |
| **SuBAC0000650** | 512 | 2309 | SES39L16 | XP_008646793 | 91.569 | 0 | PREDICTED: premnaspirodiene oxygenase-like [Zea mays]. |
| **SuBAC0000660** | 501 | 1503 | SES39L16 | XP_008646793 | 88.454 | 0 | PREDICTED: premnaspirodiene oxygenase-like [Zea mays]. |
| **SuBAC0000670** | 514 | 2106 | SES39L16 | XP_008646793 | 90.234 | 0 | PREDICTED: premnaspirodiene oxygenase-like [Zea mays]. |
| **SuBAC0000680** | 546 | 5821 | SES41F02 | XP_002449294 | 80.93 | 0 | hypothetical protein SORBIDRAFT_05g007320 [Sorghum bicolor]. |
| **SuBAC0000690** | 1060 | 5452 | SES41F02 | XP_002449293 | 90.706 | 0 | hypothetical protein SORBIDRAFT_05g007310 [Sorghum bicolor]. |
| **SuBAC0000700** | 211 | 3122 | SES41F02 | CDM81076 | 49.554 | 1.08E-62 | unnamed protein product [Triticum aestivum]. |
| **SuBAC0000710** | 567 | 9532 | SES69K24 | AGT16394 | 99.823 | 0 | sucrose-phosphate synthase [Saccharum hybrid cultivar R570]. |
| **SuBAC0000720** | 368 | 3091 | SES69K24 | AGT15921 | 98.652 | 0 | hypothetical protein SHCRBa_179_G22_F_140 [Saccharum hybrid cultivar R570]. |
| **SuBAC0000730** | 397 | 5043 | SES69K24 | AGT17003 | 98.737 | 0 | hypothetical protein SHCRBa_063_N24_F_320 [Saccharum hybrid cultivar R570]. |
| **SuBAC0000740** | 410 | 3917 | SES84H16 | XP_002456843 | 92.665 | 0 | hypothetical protein SORBIDRAFT_03g043920 [Sorghum bicolor]. |
| **SuBAC0000750** | 243 | 2297 | SES84H16 | XP_002456842 | 93.802 | 1.66E-153 | hypothetical protein SORBIDRAFT_03g043910 [Sorghum bicolor]. |
| **SuBAC0000760** | 1077 | 5434 | SES84H16 | AEO46461 | 99.349 | 0 | sucrose phosphate synthase B [Saccharum hybrid cultivar ROC22]. |
| **SuBAC0000770** | 464 | 2352 | SES84H16 | ABS89149 | 79.845 | 0 | MATE [Sorghum bicolor]. |
| **SuBAC0000780** | 76 | 228 | SES84H16 | N.A. |  |  |  |
| **SuBAC0000790** | 543 | 2519 | SES84H16 | AFW84172 | 92.939 | 0 | hypothetical protein ZEAMMB73_394344 [Zea mays]. |
| **SuBAC0000800** | 99 | 3003 | SES84H16 | NP_001148325 | 100 | 7.56E-64 | ubiquitin-like protein SMT3 [Zea mays]. |
| **SuBAC0000810** | 247 | 3212 | SES84H16 | XP_002458988 | 92.683 | 1.34E-166 | hypothetical protein SORBIDRAFT_03g043860 [Sorghum bicolor]. |
