# Supplementary material for: Comparative analysis of sucrose phosphate synthase (SPS) gene family between Saccharum officinarum and Saccharum spontaneum
Source: BMC Plant Biol. 2020 Sep 14;20:422. doi: 10.1186/s12870-020-02599-7 (PMC7488781; doi:10.1186/s12870-020-02599-7)
Supplement: Supplementary file 2 — Additional file 2. Gene identification of SPS genes in the S. spontaneum genome by Blastp with the sequence of SPS genes from BAC libraries. Two genes (Sspon.007C0001731 and Sspon.004A0021251) were reannotated. [file 12870_2020_2599_MOESM2_ESM.doc]

**Additional file 2.** Gene identification of SPS genes in *S.spontaneum* genome by BLASTp with the sequence of *SPS* genes from BAC libraries. Two genes (*Sspon.007C0001731, Sspon.004A0021270* and *Sspon.004A0021251*) were re-annotated.

| ***S.spontaneum* genome** | | | **BAC libraries** | | | **Similarity with**  **BAC sequence (%)** |
| --- | --- | --- | --- | --- | --- | --- |
| **Gene name** | **Gene ID** | **Protein Length (aa)** | **Gene name** | **BAC ID** | **Protein Length (aa)** |  |
| *SPSA* | Sspon.007C0001731 | 1043 | *SsSPSA* | SES23E05 | 944 | 96.40 |
| *SPSB* | Sspon.003B0003981 | 1081 | *SsSPSB* | SES 84H16 | 1107 | 96.96 |
| *SPSC* | Sspon.005C0019050 | 1067 | *SsSPSC* | SES 41F02 | 1060 | 91.42 |
| *SPSD1* | Sspon.008D0005891 | 749 | *SsSPSD1* | SES 32E01 | 567 | 98.68 |
| *SPSD2* | Sspon.004A0021261 | 980 | *SsSPSD2* | SES 39L16 | 956 | 93.69 |
|  | Sspon.004A0021251 | 950 |  |  |  | 95.96 |
|  | Sspon.004A0021270 | 968 |  |  |  | 95.00 |
