# Supplementary figures and images for: Comparative analysis of sucrose phosphate synthase (SPS) gene family between Saccharum officinarum and Saccharum spontaneum
Source: BMC Plant Biol. 2020 Sep 14;20:422. doi: 10.1186/s12870-020-02599-7 (PMC7488781; doi:10.1186/s12870-020-02599-7)

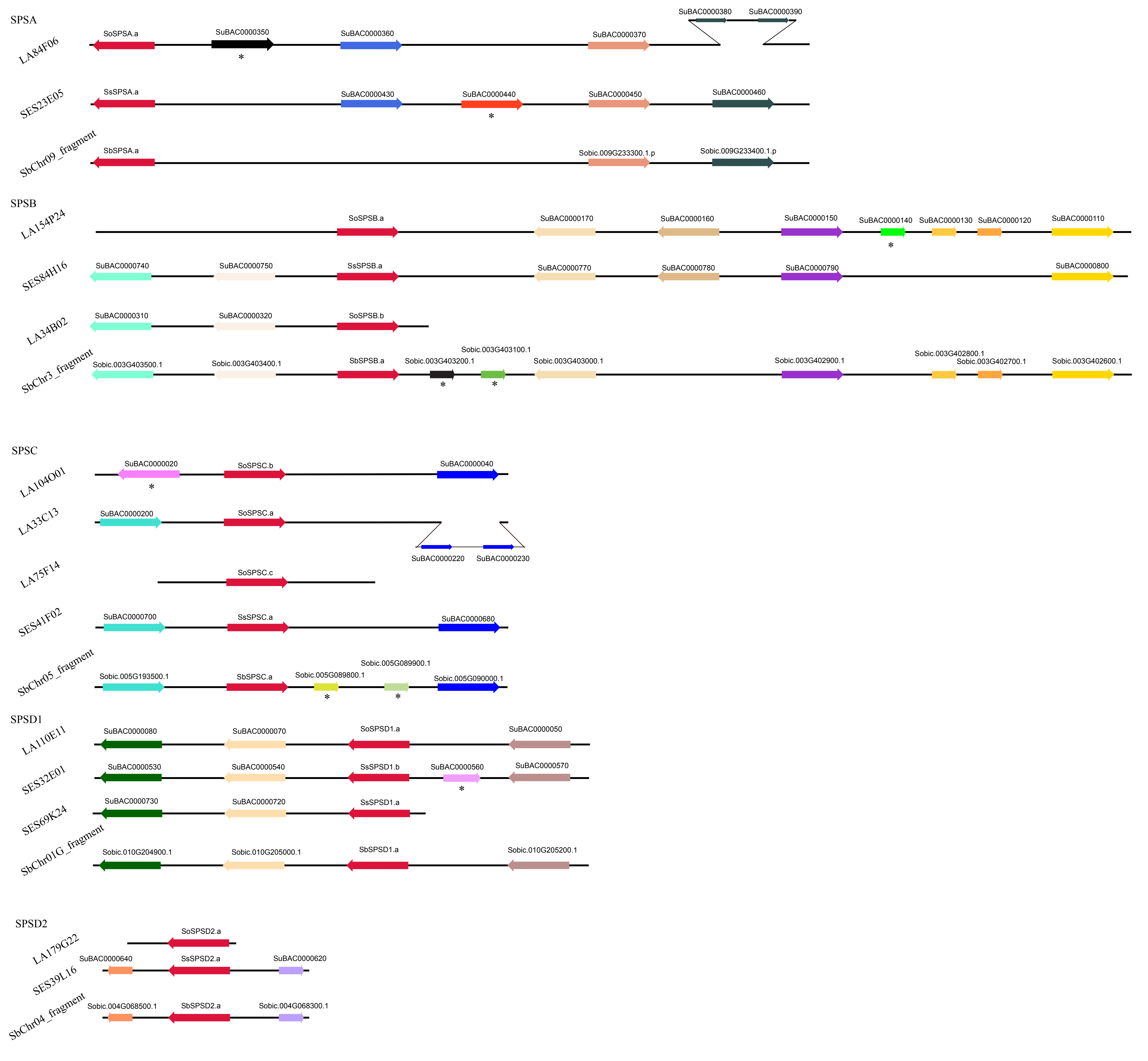

Supplement: Supplementary file 4 — Additional file 4. Syntenic comparison of genomic DNA containing SPS genes among S. officinarum, S. spontaneum and S.bicolor. Homologs are indicated by arrows with same color. The asterisks indicate single-copied genes in the selected gene set. [file 12870_2020_2599_MOESM4_ESM.pdf]

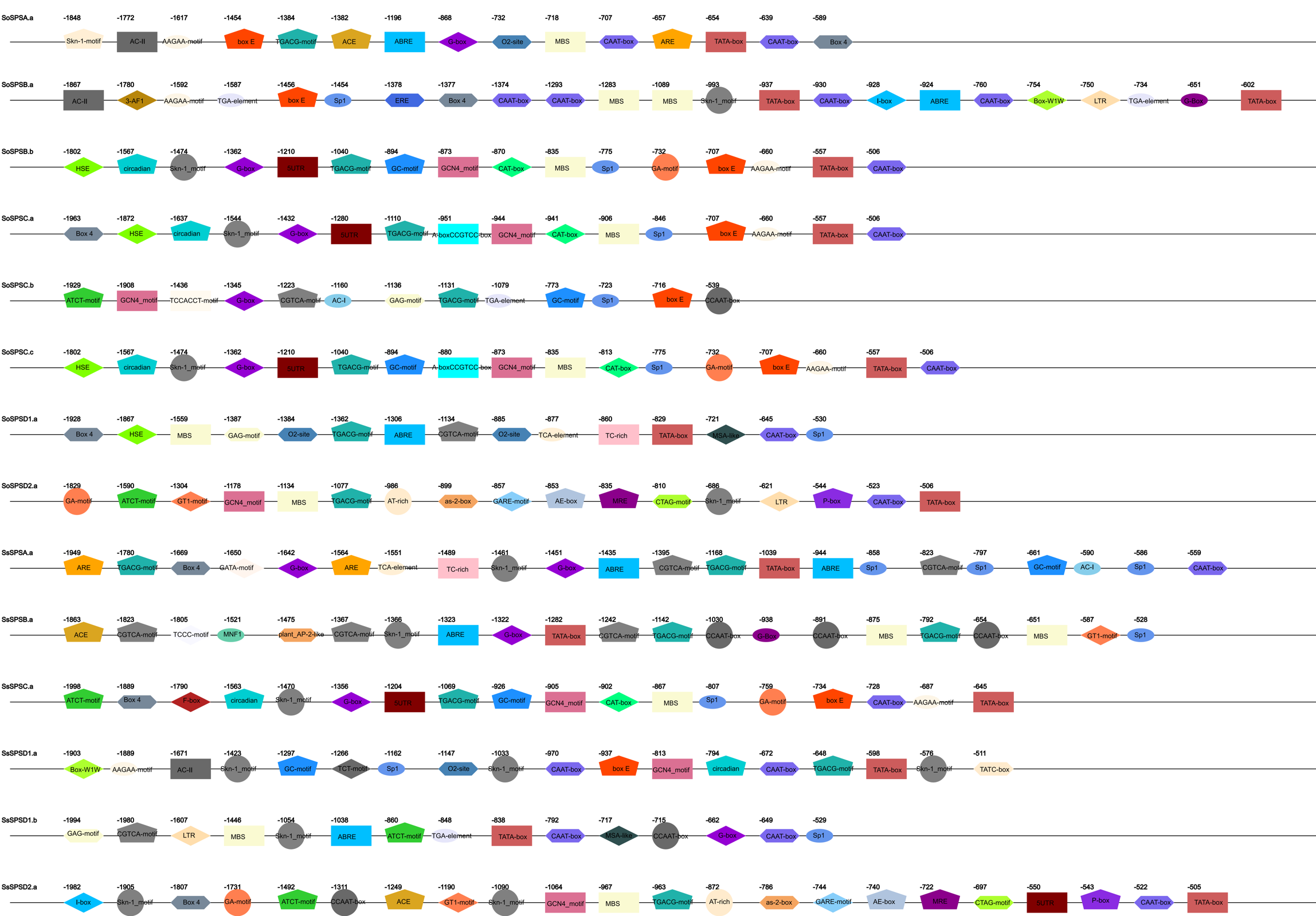

Supplement: Supplementary file 6 — Additional file 6. Cis-elements in SPS promoters. [file 12870_2020_2599_MOESM6_ESM.pdf]

A

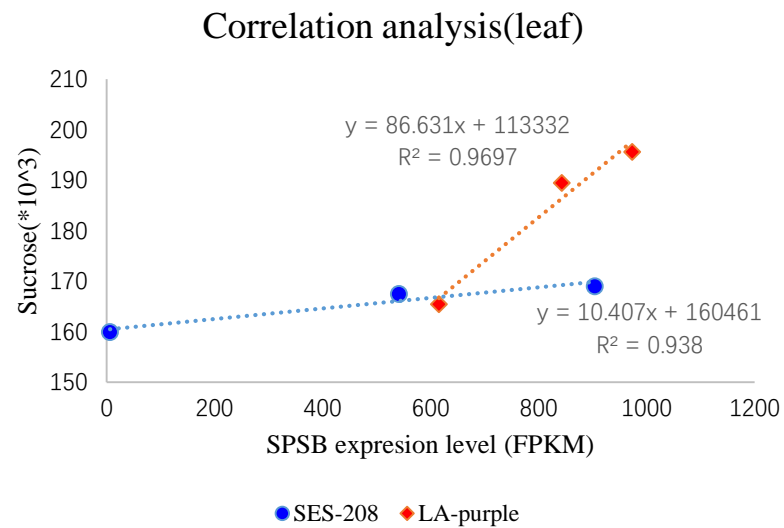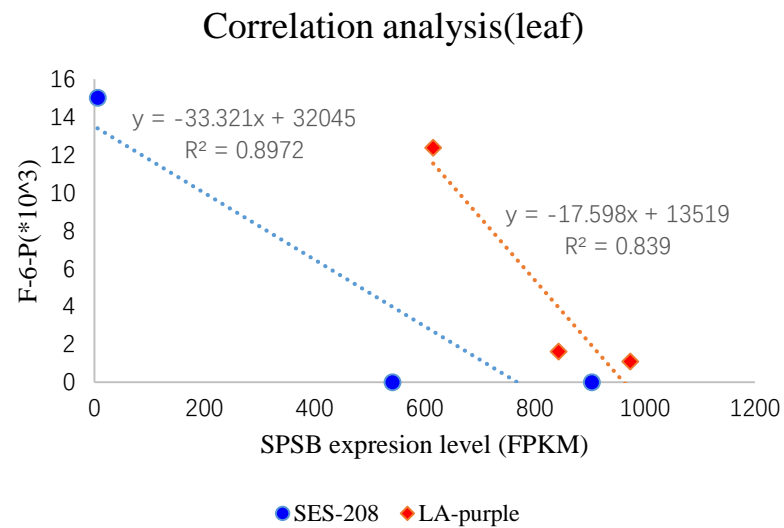

B

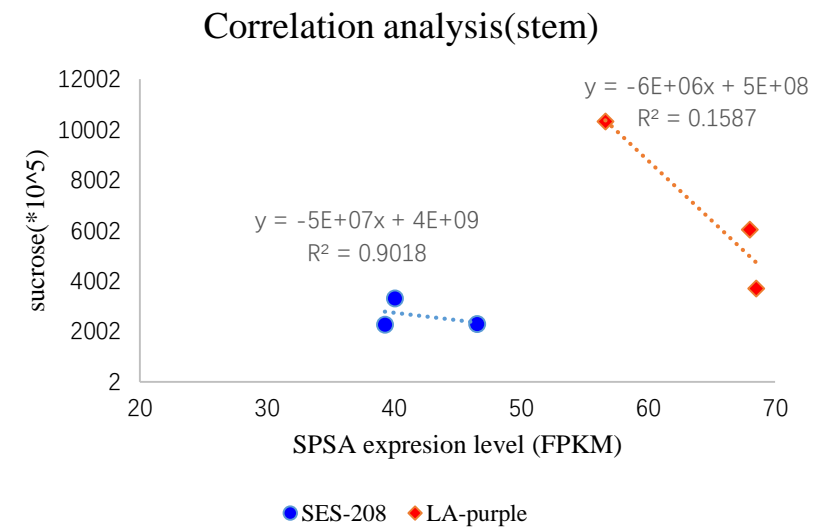

Supplement: Supplementary file 8 — Additional file 8. Correlation analysis between the expression of SPS genes and content of the metabolites in gradient developmental leaves (A, left) and stems (B, right) in Saccharum. The correlation coefficient (R2) and dependent equation of each groups data is marked near the correlation curve. R2 > 0.9 was considered to be significantly correlated. [file 12870_2020_2599_MOESM8_ESM.pdf]
