# Supplementary material for: Comparative analysis of sucrose phosphate synthase (SPS) gene family between Saccharum officinarum and Saccharum spontaneum
Source: BMC Plant Biol. 2020 Sep 14;20:422. doi: 10.1186/s12870-020-02599-7 (PMC7488781; doi:10.1186/s12870-020-02599-7)
Supplement: Supplementary file 5 — Additional file 5. Pairwise comparison of SPS homologs in Saccharum. [file 12870_2020_2599_MOESM5_ESM.doc]

**Additional file 5.** Pairwise comparison of SPS homologs in *Saccharum.*

| **Identity** | **SoSPSA** | **SoSPSB** | **SoSPSC** | **SoSPSD1** | **SoSPSD2** | **SsSPSA** | **SsSPSB** | **SsSPSC** | **SsSPSD1** | **SsSPSD2** |
| --- | --- | --- | --- | --- | --- | --- | --- | --- | --- | --- |
| **SoSPSA** | 100 |  |  |  |  |  |  |  |  |  |
| **SoSPSB** | 73.144 | 100 |  |  |  |  |  |  |  |  |
| **SoSPSC** | 65.605 | 53.105 | 100 |  |  |  |  |  |  |  |
| **SoSPSD1** | 69.978 | 34.982 | 32.917 | 100 |  |  |  |  |  |  |
| **SoSPSD2** | 73.009 | 30.605 | 27.778 | 78.535 | 100 |  |  |  |  |  |
| **SsSPSA** | 99.767 | 54.346 | 46.199 | 50.481 | 58.45 | 100 |  |  |  |  |
| **SsSPSB** | 72.926 | 99.536 | 52.754 | 34.375 | 30.743 | 54.129 | 100 |  |  |  |
| **SsSPSC** | 65.385 | 52.837 | 97.001 | 30.877 | 29.021 | 46.291 | 53.116 | 100 |  |  |
| **SsSPSD1** | 70.575 | 45.833 | 33.333 | 100 | 80.952 | 68.75 | 46.087 | 43.478 | 100 |  |
| **SsSPSD2** | 73.009 | 37.367 | 32.353 | 82.401 | 95.648 | 58.111 | 36.986 | 35.417 | 79.817 | 100 |

Note: *SPS* orthologs which shared high similarity between *S. officinarum* and *S. spontaneum* are highlighted with red.
