# Supplementary material for: Comparative analysis of sucrose phosphate synthase (SPS) gene family between Saccharum officinarum and Saccharum spontaneum
Source: BMC Plant Biol. 2020 Sep 14;20:422. doi: 10.1186/s12870-020-02599-7 (PMC7488781; doi:10.1186/s12870-020-02599-7)
Supplement: Supplementary file 7 — Additional file 7. Ka, Ks comparison of SPS orthologs/alleles in S.bicolor, S. officinarum and S. spontaneum. [file 12870_2020_2599_MOESM7_ESM.doc]

**Supplementary Table 7**. Ka, Ks comparison of *SPS* orthologs/alleles in *Sorghum bicolor*, *S. officinarum* and *S. spontaneum.*

| **Gene pair** | **Ka** | **Ks** | **Ka/Ks** | **p-value** | **Gene pair** | **Ka** | **Ks** | **Ka/Ks** | **p-value** |
| --- | --- | --- | --- | --- | --- | --- | --- | --- | --- |
| ***SbSPSA* vs *SoSPSA.a*** | 0.018739 | 0.08263 | 0.226781 | 1.41E-06 | ***SoSPSA.a* vs *SsSPSA.a*** | 0.049681 | 0.088719 | 0.559982 | 0.013003 |
| ***SbSPSA* vs *SsSPSA.a*** | 0.005844 | 0.054974 | 0.106303 | 4.56E-14 | ***SoSPSB.a* vs *SoSPSB.b*** | 0.04959 | 0.109053 | 0.45473 | 0.006698 |
| ***SbSPSB* vs *SoSPSB.a*** | 0.011179 | 0.124718 | 0.089637 | 1.36E-31 | ***SoSPSB.a* vs *SsSPSB.a*** | 0.001199 | 0.011225 | 0.106849 | 0.000509 |
| ***SbSPSB* vs *SoSPSB.b*** | 0.054948 | 0.26965 | 0.203774 | 1.03E-12 | ***SoSPSB.b* vs *SsSPSB.a*** | 0.049253 | 0.118494 | 0.415654 | 0.001533 |
| ***SbSPSB* vs *SsSPSB.a*** | 0.010802 | 0.118989 | 0.090784 | 1.92E-30 | ***SoSPSC.a* vs *SoSPSC.b*** | 0.010322 | 0.058974 | 0.17502 | 7.19E-09 |
| ***SbSPSC* vs *SoSPSC.a*** | 0.034544 | 0.334385 | 0.103305 | 2.81E-45 | ***SoSPSC.a* vs *SoSPSC.c*** | 0.014162 | 0.082972 | 0.170682 | 1.78E-06 |
| ***SbSPSC* vs *SoSPSC.b*** | 0.026586 | 0.322644 | 0.082401 | 1.06E-49 | ***SoSPSC.a* vs *SsSPSC.a*** | 0.010917 | 0.101531 | 0.10752 | 1.47E-16 |
| ***SbSPSC* vs *SoSPSC.c*** | 0.056098 | 0.454859 | 0.12333 | 4.97E-25 | ***SoSPSC.b* vs *SoSPSC.c*** | 0.027579 | 0.105449 | 0.26154 | 1.25E-05 |
| ***SbSPSC* vs *SsSPSC.a*** | 0.028831 | 0.379403 | 0.07599 | 6.12E-56 | ***SoSPSC.b* vs *SsSPSC.a*** | 0.003912 | 0.115515 | 0.033868 | 5.73E-27 |
| ***SbSPSD1* vs *SoSPSD1.a*** | 0.007626 | 0.045073 | 0.169203 | 6.36E-10 | ***SoSPSC.c* vs *SsSPSC.a*** | 0.028129 | 0.215174 | 0.130727 | 2.96E-15 |
| ***SbSPSD1* vs *SoSPSD2.a*** | 0.092353 | 0.745881 | 0.123817 | 5.70E-90 | ***SoSPSD1.a* vs *SoSPSD2.a*** | 0.09451 | 0.75361 | 0.12541 | 2.69E-89 |
| ***SbSPSD1* vs *SsSPSD1.a*** | 0.005516 | 0.048165 | 0.114521 | 2.15E-12 | ***SoSPSD1.a* vs *SsSPSD1.a*** | 0.00496 | 0.012158 | 0.408003 | 0.054591 |
| ***SbSPSD1* vs *SsSPSD1.b*** | 0.00319 | 0.042708 | 0.0747 | 5.03E-08 | ***SoSPSD1.a* vs *SsSPSD1.b*** | 0.004563 | 0.021411 | 0.213109 | 0.004794 |
| ***SbSPSD1* vs *SsSPSD2.a*** | 0.08496 | 0.720097 | 0.117984 | 1.79E-96 | ***SoSPSD1.a* vs *SsSPSD2.a*** | 0.087062 | 0.725078 | 0.120072 | 1.54E-95 |
| ***SbSPSD2* vs *SoSPSD1.a*** | 0.087472 | 0.744973 | 0.117416 | 4.71E-98 | ***SoSPSD2.a* vs *SsSPSD1.a*** | 0.093156 | 0.751814 | 0.123908 | 7.92E-90 |
| ***SbSPSD2* vs *SoSPSD2.a*** | 0.012489 | 0.058643 | 0.212961 | 1.37E-09 | ***SoSPSD2.a* vs *SsSPSD1.b*** | 0.116214 | 1.08133 | 0.107474 | 1.75E-58 |
| ***SbSPSD2* vs *SsSPSD1.a*** | 0.086186 | 0.738941 | 0.116635 | 3.70E-98 | ***SoSPSD2.a* vs *SsSPSD2.a*** | 0.006929 | 0.015565 | 0.44517 | 0.06542 |
| ***SbSPSD2* vs *SsSPSD1.b*** | 0.103314 | 1.00943 | 0.102349 | 1.24E-64 | ***SsSPSD1.a* vs *SsSPSD2.a*** | 0.085729 | 0.729513 | 0.117516 | 8.22E-97 |
| ***SbSPSD2* vs *SsSPSD2.a*** | 0.005185 | 0.047755 | 0.10857 | 1.78E-12 | ***SsSPSD1.b* vs *SsSPSD2.a*** | 0.101818 | 1.02328 | 0.099501 | 4.83E-66 |
