# Supplementary material for: Comparative analysis of sucrose phosphate synthase (SPS) gene family between Saccharum officinarum and Saccharum spontaneum
Source: BMC Plant Biol. 2020 Sep 14;20:422. doi: 10.1186/s12870-020-02599-7 (PMC7488781; doi:10.1186/s12870-020-02599-7)
Supplement: Supplementary file 9 — Additional file 9. The information for probes used to identify SPS-containing BACs. [file 12870_2020_2599_MOESM9_ESM.doc]

**Additional file 10.** Sequences of the probes that were used to identify SPS -containing BACs

| Name of gene | Forward primers | Reverse primers | Sorghum gene model |
| --- | --- | --- | --- |
| **SPSA** | CGGGGAATGACTGGATCAACA | GGTTCCAGATCCTCCACGACA | Sb09g028570 |
| **SPSB** | CAGGAAGGAACAGGAGCAGG | TCCAGGCATCATTGACATCG | Sb03g043900 |
| **SPSC** | ACGGACCCCAGGAAGAACGT | CGATCAGGGTGAGTCTGAATG | Sb05g007310 |
| **SPSD1** | ACGCAGTGACTGGATTCTCA | GCTCCCTACTGACTTTGATGC | Sb10g025240 |
| **SPSD2** | AAAACGCCAGCCAGAAACTG | AATCTCGGCCAAGCTCCATA | Sb04g005720 |
